# Influence of polyphenol-rich apple pomace extract on oxidative damage to DNA in type 2 diabetes mellitus individuals

**DOI:** 10.1186/2049-3002-2-S1-P25

**Published:** 2014-05-28

**Authors:** Annemarie Grindel, Elisabeth Müllner, Helmut Brath, Walther Jäger, Trine Henriksen, Henrik Enghusen Poulsen, Doris Marko, Karl-Heinz Wagner

**Affiliations:** 1 Department of Nutritional Sciences, University of Vienna, Vienna, Austria; 2 Diabetes Outpatient Clinic, Health Centre South, Vienna, Austria; 3 Department of Clinical Pharmacy and Diagnostics, University of Vienna, Vienna, Austria; 4 Laboratory of Clinical Pharmacology, Rigshospitalet, Copenhagen, Denmark; 5 Department of Clinical Pharmacology, Bispebjerg Hospital, Copenhagen, Denmark; 6 Health Science Faculty, University of Copenhagen, Copenhagen, Denmark; 7 Institute of Food Chemistry and Toxicology, University of Vienna, Vienna, Austria

## Background

Diabetes mellitus type 2 (DM2) is associated with increased oxidative stress and oxidative damage to DNA. An appropriate intake of antioxidants via the diet can improve this disturbed oxidative status [1]. Apples are the most widely consumed fruits in Europe and represent a major source of antioxidants due to their high polyphenol content [2]. Apple pomace as a polyphenol-rich byproduct of apple juice production could serve as a cheap and reliable tool for a nutraceutical with antioxidative properties.

## Materials and methods

To test the antioxidant potential of a pectin-depleted apple pomace extract (APE) in human subjects, a placebo-controlled, crossover, double-blind, pilot human intervention study was performed. Eighteen postmenopausal women with DM2 (age=69.7±6.7 y; BMI=33.9±4.5 kg/m2) were randomly allocated to receive either APE (440 mg per capsule containing about 100 mg total polyphenols, once daily) or placebo during two 4-week supplementation periods separated by a 4-week wash-out period. Before and after each supplementation period oxidative damage to DNA (Comet Assay) in peripheral blood mononuclear cells (PBMC) and whole blood, urinary excretion of 8-oxo-7hydro-2’-deoxyguanosine (8-oxodG) and 8-oxo-7,8-dihydroguanosine (8-oxoGuo), glycated hemoglobin (HbA1c), fasting blood glucose, insulin, C-peptide and anthropometric indices were measured. The bioavailability of the main APE polyphenol Phloridzin and its metabolite Phloretin were analyzed in plasma samples.

## Results

In contrast to the placebo-supplementation, APE resulted in detectable plasma Phloridzin (12.7±40.7 ng/ml) and Phloretin (19.3±36.5 ng/ml) concentrations. The study population was characterized by HbA1c =5 4.9±6.3 mmol/mol, fasting blood glucose = 8.1±1.9 mmol/l, fasting insulin = 99.3±36.6 pmol/l and C-peptide = 1.3±0.4 nmol/l baseline levels. However, these DM2 biomarkers were not influenced by the supplementation with APE compared to placebo. No changes occurred in 8-oxoGuo and 8-oxodG. FPG-sensitive sites of whole blood decreased (*P* = 0.026) regarding apple pomace intervention of both diet periods. Neither DNA strand breaks nor H_2_O_2_-sensitivity of DNA altered following APE supplementation.

## Conclusions

Oxidatively damaged purines decreased after APE intervention while other markers of oxidative damage to DNA in DM2 individuals did not change after short-term supplementation with polyphenol-rich APE.
